# Neural network analysis of the contribution of psychotropic prescription sequences to the risk of non-psychiatric adverse events in bipolar and schizophrenia spectrum disorders

**DOI:** 10.3389/fdgth.2025.1633220

**Published:** 2025-09-04

**Authors:** Nathan Vidal, Mohammed Sedki, Nadia Younès, Hugo Bottemanne, Paul Roux, Eric Brunet-Gouet

**Affiliations:** ^1^ Centre Hospitalier de Versailles, Service Universitaire de Psychiatrie d’Adultes et d’Addictologie, Le Chesnay, France; ^2^ DisAP, MOODS Team, INSERM UMR1018, CESP, Université de Versailles Saint-Quentin-En-Yvelines—Université Paris-Saclay, Le Chesnay, France; ^3^ OncoStat Team, INSERM UMR1018, CESP, Institut Gustave Roussy, Université Paris-Saclay, Villejuif, France; ^4^ DevPsy-CESP, INSERM UMR1018, Université Paris-Saclay, Université de Versailles Saint-Quentin-En-Yvelines, Villejuif, France; ^5^ MOODS team, INSERM UMR1018, CESP, Faculté de Médecine Paris-Saclay, Université de Versailles Saint-Quentin-En-Yvelines, Université Paris-Saclay, Villejuif, France; ^6^ Department of Psychiatry, Bicêtre Hospital, Mood Center Paris Saclay, DMU Neurosciences, Paris-Saclay University, Assistance Publique-Hôpitaux de Paris (AP-HP), Kremlin-Bicêtre, France; ^7^ Service de Psychiatrie du Secteur 78G18, Centre Hospitalier de Plaisir, Plaisir, France

**Keywords:** schizophrenia, bipolar disorders, psychotropic drugs, adverse drug event, psychotropic adverse effects

## Abstract

Psychotropic medications are associated with lower mortality in bipolar disorders (BD) and schizophrenia spectrum disorders (SZD) but may trigger serious adverse events requiring hospitalization. Determining the iatrogenic causes of such events can considerably help psychiatrists understand their development and adjust the prescription accordingly. We aimed to assess to what extent the psychotropic prescription sequence contributes to in-hospital non-psychiatric adverse events in BD and SZD. We conducted a case-control design including adults with BD or SZD from the French national healthcare system claims database (*n* = 87,182). A recurrent neural network model was trained to discriminate between adults who experienced adverse events and matched adults who did not, based only on psychotropic prescription sequences over the past 18 months and demographic data. Explainable AI combined enabled us to understand the model's prediction. Psychotropic doses during the months preceding the adverse events were relatively more important than earlier doses to predict in-hospital urinary retention and thyroid disorders, but it was not the case to predict movement or cardiac disorders. The doses of certain benzodiazepines, tropatepine, quetiapine, clozapine, loxapine, lithium salts, and valproate were significant risk factors for adverse events. A recurrent neural network combined with explainable AI identified key psychotropic prescription features and duration associated with non-psychiatric adverse events among a large number of features. Yet, it was unable to predict events with high accuracy. Such a model could only be used retrospectively to generate hypotheses about iatrogenic risk factors for adverse events, offering limited value for integration into prescription softwares.

## Introduction

Bipolar disorders (BD) and schizophrenia spectrum disorders (SZD) are typically treated by psychotropic medications to prevent acute episodes in inpatient and outpatient care. Psychotropic medications are associated with lower all-cause mortality in SZD (1) and BD (2), but may lead to adverse drug events (ADEs), ranging from mild discomforts to serious medical emergencies that require hospitalization, such as hyponatremia or delirium. Non-psychiatric in-hospital adverse events affect half of inpatients with BD or SZD and are associated with longer hospitalizations (3). A first ADE significantly increases the risk of recurrence (4), highlighting the need to identify its iatrogenic causes. ADEs leading to hospitalization in young adults with BD or schizoaffective disorders are primarily attributed to lithium salts and second-generation antipsychotics (5). While anxiolytics and sedatives account for the highest absolute number of emergency department visits for ADEs, antipsychotics and lithium salts are the most frequently associated with emergency department visits for ADEs when adjusted for prescription prevalence (6). High psychotropic doses (7), psychotropic polypharmacy (5), and prolonged treatment duration (8) are also significantly associated with higher risks of in-hospital adverse events in BD and SZD. Conversely, serious ADEs may emerge after the introduction of a new medication regardless of dosage (9). While some adverse events are typically associated with specific medications (e.g., serotonin syndrome with antidepressants), others like delirium, have multiple putative iatrogenic causes, further complicating prevention. Identifying the prescription practices most strongly associated with ADEs with multiple putative iatrogenic causes could help clinicians understand their development, identify at-risk patients, and adjust prescriptions accordingly.

Studies investigating the non-psychiatric adverse events associated with psychotropic prescription practices using claims data (10) or pharmacovigilance databases (11) commonly apply traditional frequentist methods (e.g., logistic regression, survival analysis) to provide directly interpretable risk estimators as odds or hazard ratios. However, these models assume linear associations between predictors and risk of adverse events, rely on predefined model structures, and require excluding certain patients based on design constraints. For instance, patients with very stable prescriptions, common among outpatients with BD and SZD, are uninformative in case-crossover designs (12). Additionally, the risk of adverse effects may depend on the duration of psychotropic prescriptions (13), a feature rarely addressed in pharmacoepidemiological studies. A model capable of processing long-term sequences of psychotropic dosages could identify among the large number of prescription features the ones most associated with adverse events in naturalistic contexts and suggest when the risk is the highest.

Machine learning methods have emerged as powerful tools for handling a large number of variables and identifying non-linear associations. Most machine learning models designed for pharmacy or pharmacology were trained to predict new drugs' adverse reactions at the early stage of the drug development (14). A few models exhibited good performance to reliably predict adverse reactions in the context of non-psychotropic medications, like in the case of prescription in newborns (C-statistic = 0.91) (15) or hospitalized patients with chronic kidney disease (C-statistic = 0.81) (16). Besides, most predictive models designed for clinical psychiatry estimated the risk for psychiatric outcomes such as suicide attempts or relapse, or future psychotropic prescriptions (17). To our knowledge, no machine learning model estimated the risk for non-psychiatric adverse events in the context of psychiatric care. Recurrent Neural Networks (RNNs) have been effectively applied to various medical tasks involving time-series, with demonstrated effectiveness in predicting next-period prescriptions by learning from patients' history (16). One type of RNN, Gated Recurrent Units (GRU) (17), effectively handles sequential data with temporal dependencies while mitigating vanishing or exploding gradients, which can hinder the ability of RNNs to learn long-range dependencies. GRU could be relevant to reveal the complex associations between multiple long-term sequences of psychotropic dosage and adverse events.

A key limitation of machine learning models compared to traditional frequentist methods in public health is the lack of transparency on the relationship between input features and predictions, i.e., the “black box” nature (18). Explainable AI (XAI) methods have emerged to clarify the rationale behind the model's decision (19). Applying XAI to a machine learning model can improve clinicians' understanding and trust in the model's prediction. Besides, sequences of psychotropic prescriptions are often available in structured formats within prescription softwares or as pharmacy deliveries, facilitating automated processing. A model capable of providing valid risk estimates of ADEs based on such data could be valuable for clinical settings.

Our objective was to develop a GRU-based model that discriminates between patients with BD or SZD who experienced in-hospital adverse events and matched patients who did not, based solely on psychotropic prescription sequences and demographics. By incorporating explainable AI methods we aimed to identify the psychotropic drugs, doses, and treatment durations most associated with the development of in-hospital non-psychiatric adverse events. Our goal was not to reliably and accurately predict in-hospital adverse events, but rather evaluate the contribution of psychotropics to such events among patients with BD or SZD, identifying the prescription features clinicians should monitor or investigate in priority, and assess the relevance of implementing such a model in a prescription software, where only medication data are available, and not clinical test results, procedures, or comorbidity information.

## Methods

Analyses were conducted on *SAS*® Enterprise Guide v7.4 (SAS Institute North Carolina, USA) and Python.

### Participants

We conducted a case-control nationwide longitudinal study, including patients from the French National Health Data System (SNDS), which covers medical interventions, hospitalizations, and outpatient treatments for 99% of the French population (over 65 million people). As the data is pseudonymised, patient consent was not required. We included individuals included in the SNDS from January 1, 2013 to December 31, 2022 with schizophrenia spectrum (SZD) and/or bipolar disorders (BD) defined as:

1. Patients receiving 100% reimbursement for long-term conditions within the year, with diagnosis codes F20–25, F28, or F29 for SZD, and F30–31 for BD according to the international classification of diseases—10th revision (ICD-10) (Supplementary Table S1).
2. Patients with at least one hospitalization for SZD or BD (as primary or related diagnosis) within the previous two years.
3. Patients with at least one hospitalization for SZD or BD within the previous five years, combined with at least three purchases of specific medications within the year:
  - For SZD: antipsychotics (ATC code N05A, excluding N05AN).
  - For BD: antipsychotics, lithium, valproate, carbamazepine, oxcarbazepine, or lamotrigine.

We excluded patients with missing data for age or sex, patients under 18 or over 65 years old, and twins or other multiple births (due to indistinguishable hospital records).

### Outcome

We identified in-hospital adverse events recognized as psychotropic drug adverse events in the literature (20) that were:

- Mostly acute, with severity developing over a short period (weeks to a couple of months).
- Severe enough to be reported in hospitalization.
- Reversible: if drug-induced, the condition resolves or becomes less severe after drug discontinuation.
- Not rare, defined as having a frequency >0.1%. In our sample, this corresponded to events reported by more than 885 individuals.

Selected in-hospital adverse events were urinary retention, constipation, cardiac rhythm or conduction disorders, electrolyte imbalances, pneumonia, seizures, delirium spectrum disorders, thyroid disorders and motor disorders (Supplementary Table S2). We included patients who experienced an outcome event at least 18 months after their initial BD or SZD diagnosis to exclusively study psychotropic prescriptions for those disorders. We only studied the first hospitalization with an adverse event after the initial diagnosis of BD or SZD.

### Medications

We collected dispensations of antidepressants (ATC code starting by N06A), anxiolytics, hypnotics, lithium salts and antipsychotics (N05), antiepileptics (N03A), and antiparkinsonians with anticholinergic properties (N04A), based on outpatients' reimbursement records, for the 18 months preceding the event. Only medications purchased before the event were collected. As information on inpatient psychotropic prescription is missing in the SNDS, we provided the model with a variable coding the occurrence of hospitalization (psychiatric or non-psychiatric) over the month so that the model could account for the possible lack of information on treatment.

We defined the duration of a prescription as the time interval between two consecutive treatment deliveries, based on the assumption that individuals who purchased a medication again had fully consumed their previous supply (see Supplementary Material 1). We did not apply a strict definition of treatment duration, as it is not appropriate for psychotropic medications, which are frequently discontinued due to factors like forgetfulness, adverse effects, or intermittent symptoms. We computed the mean daily dose over the month for each medication and each of the 18 months preceding the event. Artefactual null doses may appear when a patient does not purchase medication every month. For instance, if a patient purchases two boxes in one month and none the next month, it would appear as if they took a high dose of the treatment before discontinuing it. Therefore, we smoothed the doses by applying a sliding weighted temporal average (21), using the formula:

$$D_{i,d,m}^{\prime }=\frac{\left(D_{i,d,m-1}+\;4\times D_{i,d,m}+D_{i,d,m+1}\right)}{6}$$

with *D_i,d,m_*, the dose of the drug d for individual i at month m

### Statistical analyses

#### Matching

We matched case individuals to control individuals hospitalized in the same type of ward (psychiatric or non-psychiatric) during the month of the outcome event but who did not experience any adverse event. Matching criteria included sex, age as of 18 months prior to the event, diagnoses of BD, SZD, traumatic brain injury, Parkinson's disease, dementia, multiple sclerosis, epilepsy, cerebrovascular disease, or intellectual disability recorded between January 1, 2013 and the date of the adverse event. We also matched individuals on frailty, assessed by the Charlson Comorbidity Index over the year of the adverse event (22) (see Supplementary Table S3 for the definitions of comorbidities).

#### Preprocessing variables

We normalized the mean daily dose and age. Other predictors were binary coded: hospitalization over the month, sex (0 for males), BD and SZD diagnoses. Besides, the nine outcome variables (i.e., adverse events) were binary coded and were not mutually exclusive: several adverse events could be reported during the same hospitalization.

#### Classification models

Each model took as input the 18 normalized mean daily doses for each medication and indicators of hospitalization (i.e., one measure every month), BD and SZD diagnoses, age at inclusion, and sex. Each model returned a 9-dimension vector representing the predicted probabilities of the nine adverse events (Supplementary Table S2).

**Figure 1:**
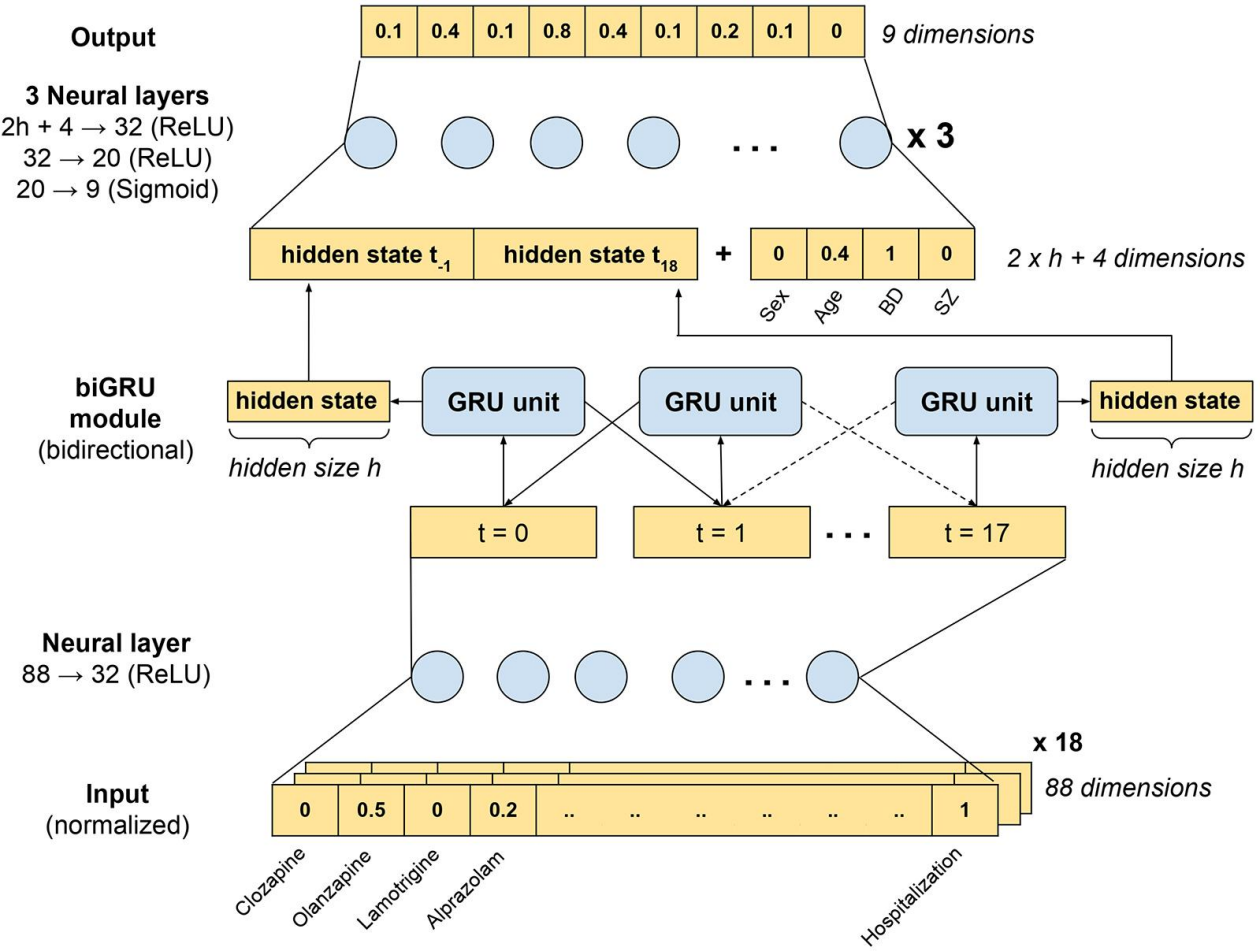
Representation of the biGRU-based model. The input was an 88 × 18 matrix (i.e., 88 variables measured over 18 months) containing normalized psychotropic drug dosages and past hospitalizations. This input was first processed by one neural layer that reduced the number of dimensions from 88 to 32, compressing the largely sparse input. The resulting 32 × 18 matrix was then processed by a biGRU module to detect time patterns. The GRU module processed the sequence onward and backward. We collected the two final hidden states (i.e., h-sized vectors), which were concatenated with a vector containing time-independent variables (sex, age at inclusion, BD and SZ diagnoses). The resulting vector of dimension (2 h + 4) was then reduced to a 9-dimension vector through three successive neural layers. A sigmoid activation function was applied to produce a final output with values varying between 0 and 1, interpreted as probabilities.

We built a model combining a bidirectional GRU and traditional feedforward neural layers, referred to as the bidirectional GRU or biGRU-based model (Figure 1). The input was a 88 × 18 matrix (i.e., 88 variables measured over 18 months) including only time-dependent variables (normalized medication dosages and hospitalization). The input matrix was first processed by one neural layer to reduce the dimensions from 88–32 before being fed to the biGRU module. A GRU unit uses an input gate, an output gate, and a forget gate to regulate the flow of past (previous month) and new information (current month), allowing the model to retain relevant past information over arbitrary time intervals (23). A biGRU module processes the input iteratively onward (from the first to the last month) and backward (from the last to the first month), reducing the risk of forgetting the features of the first month (i.e., vanishing gradient). The hidden states of the GRU unit, which are vectors representing what the module memorized from the sequence, were collected after the onward and backward processing. Those hidden states were concatenated together and with a vector containing time-independent variables (sex, age at inclusion, BD and SZD diagnoses). The concatenated vector was then reduced and modified by three traditional neural layers to produce a nine-dimension vector with values varying between 0 and 1, corresponding to the event probabilities. The size of the hidden state of the GRU unit was a hyperparameter, meaning we selected its value based on performance of the model in the train subset.

The biGRU-based model had 5,601 trainable parameters. Initial parameters were randomly selected. Then, for each trial (also called epoch), the parameters of the model were corrected, depending on the learning rate, to minimize the prediction loss measured by the loss function. We used a weighted version of the cross entropy loss function: like any loss function, it measured the error between the true label and the output, but amplified the error when the event occurred. This method counteracts the fact that a machine learning model tends to predict the most common event when outputs are unbalanced. The weights were defined as the reversed frequency of the event in the sample: 0.9418 for urinary retention, 0.8540 for constipation, 0.8873 for electrolytic disorders, 0.9742 for motor disorders, 0.9487 for delirium spectrum disorders, 0.9753 for seizures, 0.9096 for pneumonia, 0.9694 for cardiac rhythm or conduction disorders, and 0.9259 for thyroid disorders. We used the rectified linear function (ReLU) as activation function after each neural layer except after the output layer where we used the sigmoid function to produce values between 0 and 1. Adam optimizer was used to optimize the gradient descent, i.e., the correction applied to the trainable parameters, with a 0.0005 learning rate and 128 batch size. Dropout was set to 10% to reduce the risk of overfitting.

We compared this model with more explainable classification models: Random Forest (RF) and Extreme gradient boosting (XGB) (see Supplementary Material 2).

#### Training and selection of hyperparameters

**Figure 2:**
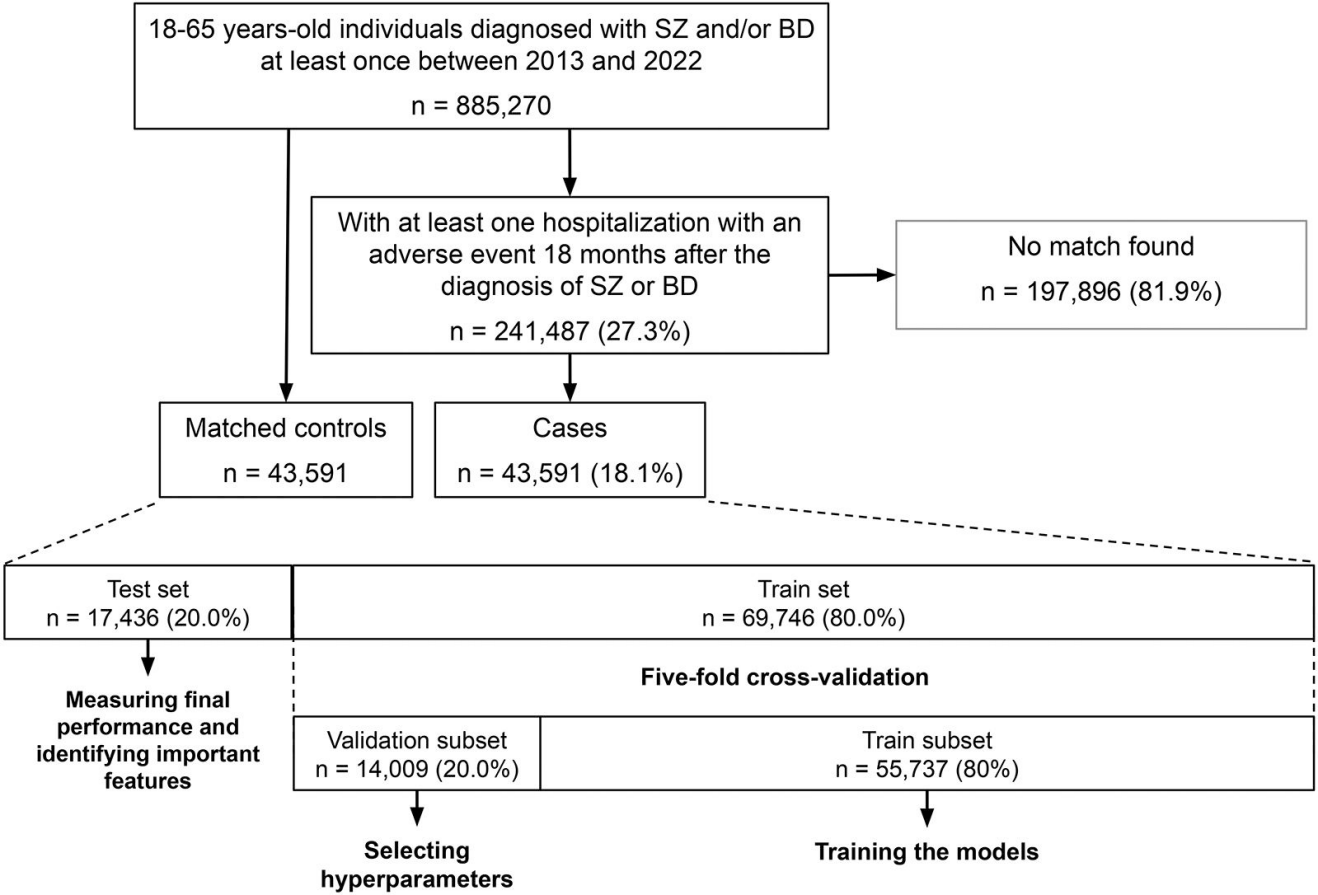
Flowchart of the study. Initially, 885,270 adults with SZD or BD aged between 18 and 65 years-old were identified between 2013 and 2022. Among those, 241,487 received a diagnosis code for an in-hospital adverse event at least 18 months after the initial SZD or BD diagnosis. We matched 43,591 of those individuals to 43,591 control individuals recruited from the initial sample. Matching criteria included age, sex, BD diagnosis, SZD diagnosis, neurological comorbidities, and frailty level. The sample of control and case individuals was then split into one train set (80%) and one test set (20%) to measure the final performance of the model. During cross-validation, the train set was split into a train subset (80%) to train the model, and a validation subset (20%) to identify the hyperparameters yielding the best performance.

As recommended (24), models were trained on the same training set, which constituted 80% of the initial dataset (Figure 2). To mitigate overfitting of the biGRU-based model and improve generalizability of the results, we trained the model on a subset of the training set and evaluated the prediction loss on a separate validation subset after each epoch. Training was interrupted when the loss in the validation subset stopped decreasing, i.e., when learning stopped improving, for 15 consecutive epochs, thereby reducing the risk of overfitting. The final model was selected based on the lowest prediction loss observed in the validation subset.

Hyperparameters were selected through a five-fold cross-validation process, which consisted in splitting five times the training set into a training and a validation subset using an 80/20 split. We trained the models in the training subset and measured their performances with the area under the receiver operating characteristic curve (AUC) in the associated validation subset for each hyperparameter value. We then averaged the AUC over the five splits and selected the set of hyperparameters resulting in the highest mean AUC.

#### Testing the models

To identify the best-performing model and estimate the predictive power achievable when considering only psychotropic prescription sequences and demographic data, we evaluated the performance of the models in the test set, i.e., the remaining 20% of the initial dataset. The most performant model was selected to identify the most relevant contributors to the prediction.

#### Explaining the predictions

XAI methods aim to explain the output of a model and have been commonly used to improve the explainability of neural network models, which are not directly interpretable (24). SHapley Additive exPlanations (SHAP) values can be used to assess the contribution of input features to the output of a machine learning model (19). We computed SHAP values in the test set using the Python package *shap*. To measure SHAP values, each feature value of a patient is considered a “player” in a cooperative game and the model's prediction represents the “payout”. SHAP values estimate how to distribute this payout among the features of each patient, thereby providing insight into their contribution to the model's predictions. In the case of a neural network, SHAP values correspond to the expected gradient-based attribution (25), which is the difference between the current gradient and the gradient applied to the model using sampled reference inputs from the background dataset. A SHAP value different to 0 indicates that the feature value contributes more to the gradient than the feature reference value. A positive SHAP value indicates the feature value contributes to increase the output value, i.e., the predicted probability of adverse event, whereas a negative SHAP value indicates the feature value contributes to decrease the predicted probability of adverse event. SHAP values were converted into percentages of the total individual SHAP value to obtain individual relative measures of the features' importance, enabling comparisons between individuals. We then averaged SHAP values across time or features to assess their average contributions to the model's predictions.

#### Estimating the associated risks

To assess the robustness of the results obtained with SHAP and provide relevant risk estimates of adverse events for clinical settings, we conducted logistic regression analyses conditioned on matched control patients, thereby controlling for matching variables. Predictors included the medications ranked among the top 10 features with the highest absolute SHAP values.

## Results

### Description of the sample

The final sample included 87,194 individuals with BD or SZD (53.7% females, aged 46.5 ± 11.3) (Table 1). Among them, 26,508 (30.4%) individuals had only BD diagnoses, 43,376 (49.8%) had only SZD diagnoses, and 17,298 (19.8%) had both BD and SZD diagnoses. We collected the dosages of 87 different psychotropic medications.

**Table 1:** Description of the sample at the month of the event. Individuals with a discharge diagnosis of adverse events were matched with control individuals hospitalized in the same type of ward (psychiatric or non-psychiatric) during the same month without any adverse event discharge diagnosis. Matching variables were highlighted in grey.

Category		With an adverse event		Without adverse event	
		(*n* = 43,591)		(*n* = 43,591)	
		mean (SD)	*n* (%)	mean (SD)	*n* (%)
Female, *n* (%)		-	23,420 (53.7%)	-	23,420 (53.7%)
Age, mean (SD)		46.5 (11.3)	-	46.5 (11.3)	-
Bipolar disorders, *n* (%)			21,903 (50.2%)	-	21,903 (50.2%)
Schizophrenia spectrum disorders, *n* (%)		-	30,337 (69.6%)	-	30,337 (69.6%)
Hospitalized over the month in the psychiatric ward, *n* (%)		-	14,642 (33.6%)	-	14,642 (33.6%)
Charlson Comorbidity Index, mean (SD)		0.79 (1.28)	-	0.79 (1.28)	-
Individuals with a non-zero Charlson Comorbidity Index, *n* (%)		-	19,582 (44.9%)	-	19,582 (44.9%)
Traumatic brain injury		-	453 (1%)	-	453 (1%)
Parkinson's disease		-	57 (0.1%)	-	57 (0.1%)
Dementia		-	53 (0.1%)	-	53 (0.1%)
Multiple sclerosis		-	17 (<0.1%)	-	17 (<0.1%)
Epilepsy		-	339 (0.8%)	-	339 (0.8%)
Cerebrovascular disease		-	346 (0.8%)	-	346 (0.8%)
In-hospital adverse events	Urinary retention, *n* (%)	-	5,122 (11.8%)		-
	Constipation, *n* (%)	-	12,835 (29.4%)	-	
	Electrolyte imbalances, *n* (%)	-	9,822 (22.5%)	-	
	Motor disorders, *n* (%)	-	2,269 (5.2%)	-	
	Delirium spectrum disorders, *n* (%)	-	4,874 (11.2%)	-	
	Seizures, *n* (%)	-	2,114 (4.8%)	-	
	Pneumonia, *n* (%)	-	7,961 (18.3%)	-	
	Cardiac rhythm or conduction disorders, *n* (%)	-	2,553 (5.9%)	-	
	Thyroid disorders, *n* (%)	-	6,283 (14.4%)	-	

### Performance of the models

Training of the biGRU-based model is illustrated in Supplementary Figure S1. We selected the hyperparameters yielding the best performance (Supplementary Table S4). The most performant model to predict adverse events was the biGRU-based model (mean AUC = 0.60), followed by the XGB model (mean AUC = 0.56) (Supplementary Table S5). The RF model performed no better than randomness. Only the biGRU-based model exhibited a balanced trade-off between sensitivity and specificity for all outcome variables.

### Iatrogenic risk factors of adverse events

To explain the predictions of the biGRU-based model, we measured the SHAP values associated with each feature of the input matrix. To facilitate interpretations, we illustrated how SHAP values explain the final prediction of urinary retention for one individual (Supplementary Figure S2).

**Figure 3:**
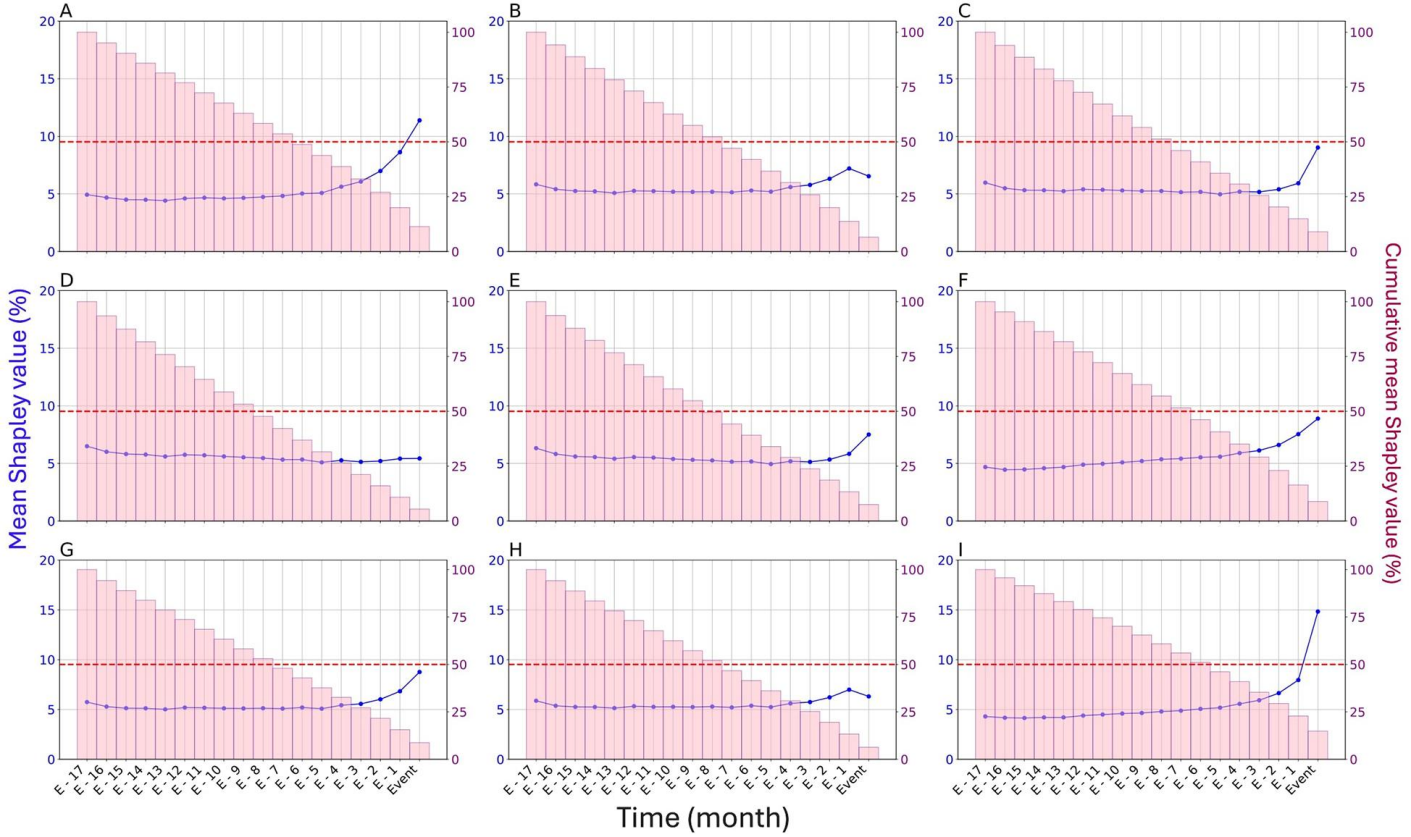
Relative importance of time in the prediction of adverse events by the biGRU-based model in the test set. **(A)** Urinary retention, **(B)** Constipation, **(C)** Electrolyte imbalances, **(D)** Motor disorders, **(E)** Delirium spectrum disorders, **(F)** Seizures, **(G)** Pneumonia, **(H)** Cardiac rhythm or conduction disorders, **(I)** Thyroid disorders. The relative importance of time was assessed by averaging SHAP values across months (displayed in blue). Cumulative mean SHAP values for the current month and all subsequent months leading up to the event are represented by pink bars. These cumulative SHAP values indicate the sequence duration necessary for the model to make its prediction: when the cumulative mean SHAP value exceeds 50% of the total SHAP value (identified by the red dotted line), the information from that month onward was, on average, sufficient for the model to reach a decision.

To assess the role of time in model predictions, we averaged SHAP values over each timepoint (Figure 3). SHAP values were on average higher over the few months directly preceding urinary retention, electrolyte imbalances, pneumonia, seizures, delirium spectrum disorders, and thyroid disorders, suggesting that the months directly preceding these events were relatively more important for the model. More than half of the model's prediction of urinary retention, seizures and thyroid disorders was made only by considering the seven or eight months preceding the event.

**Figure 4:**
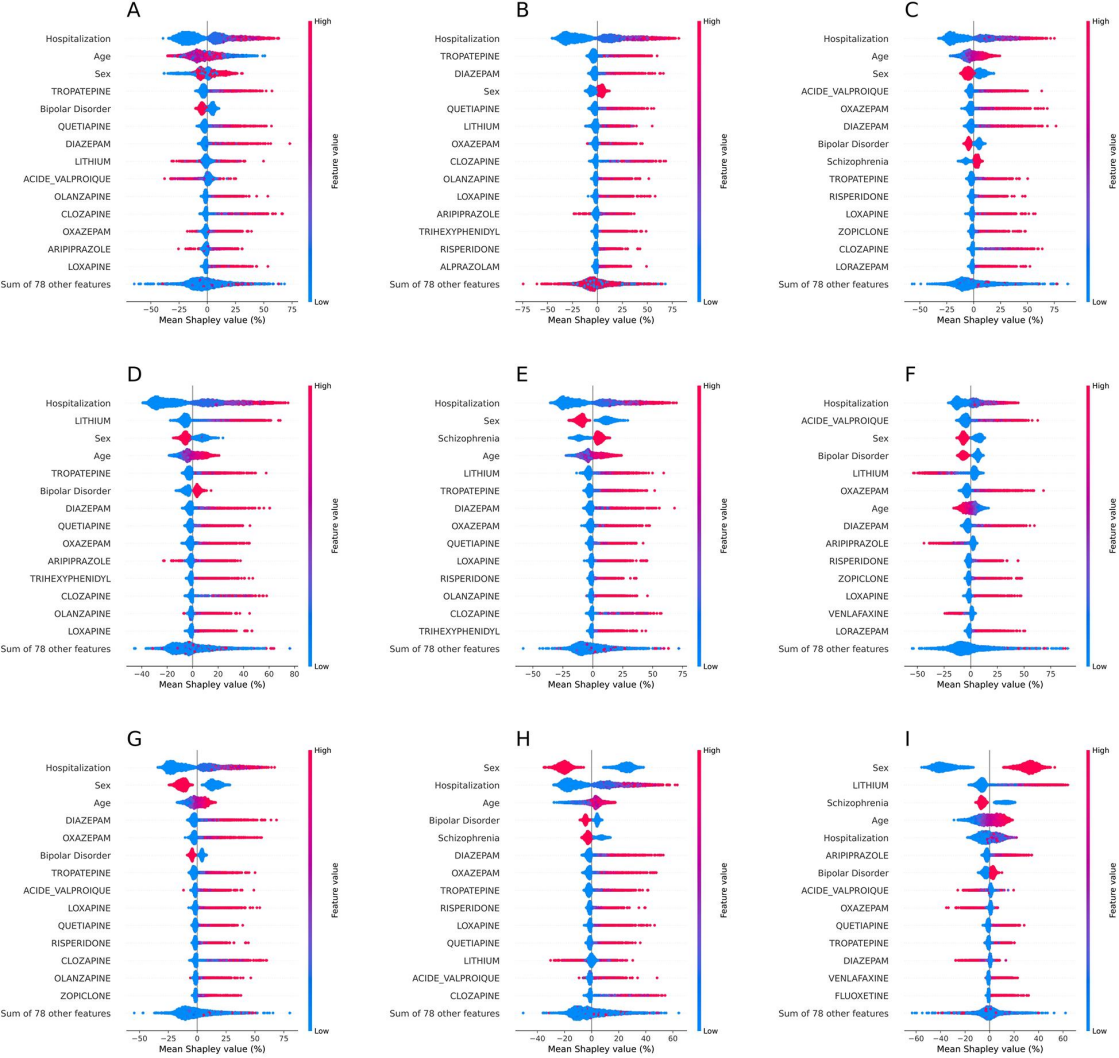
Relative importance of feature values in the prediction of adverse events by the biGRU-based model in the test set. **(A)** Urinary retention, **(B)** Constipation, **(C)** Electrolyte imbalances, **(D)** Motor disorders, **(E)** Delirium spectrum disorders, **(F)** Seizures, **(G)** Pneumonia, **(H)** Cardiac rhythm or conduction disorders, **(I)** Thyroid disorders. The relative importance of features was measured by averaging SHAP values across features. Features are listed from top to bottom by mean absolute SHAP value. Only the 14 features with the highest absolute SHAP values are displayed. A positive SHAP value indicates that the value of the feature encouraged the prediction of the adverse event, while a negative SHAP value indicates the value of the feature encouraged the prediction of its absence. One dot represents one patient and its colour indicates the feature value for that patient.

To more accurately explain the model's predictions, we calculated the mean individual SHAP values associated with each feature over the whole period. Being hospitalized over the 18 months before the event was considered the most prominent factor to predict all adverse events except cardiac and thyroid disorders (Figure 4). Past hospitalizations of certain patients had SHAP values higher than 50%, therefore contributing to more than half of the decision for that patient. Age and sex were also among the most influential features. Older age favored the prediction of electrolyte imbalances, motor disorders, delirium spectrum disorders, pneumonia, cardiac disorders and thyroid disorders, whereas younger age favored the prediction of seizures. Being a woman greatly favored the prediction of thyroid disorders, and being a man greatly favored the prediction of cardiac disorders and pneumonia.

The doses of diazepam, oxazepam, tropatepine, quetiapine, olanzapine, valproate, and lithium salts over the 18 months before the event were key factors to predict adverse events. For instance, the doses of tropatepine, quetiapine and diazepam most favored the prediction of urinary retention and constipation. We noticed positive associations between SHAP values and medication doses, suggesting some predictions were dose-dependent. The high doses of certain psychotropics contributed to more than half of the model's decision for certain patients.

### Estimating the iatrogenic risks of adverse events

We quantified the risk for adverse events associated with psychotropic doses using conditional logistic regression analyses. Diazepam dose at the month of the event was significantly associated with higher risks of any adverse event except thyroid disorders (1.02 ≤ OR ≤ 1.04 mg/day) (Supplementary Table S6). Oxazepam dose was significantly associated with higher risks of all adverse events, except urinary retention and thyroid disorders (1.03 ≤ OR ≤ 1.05/10 mg/day). Tropatepine dose was significantly associated with higher risks of urinary retention, constipation, electrolyte imbalances, motor disorders and delirium spectrum disorders (1.01 ≤ OR ≤ 1.05 mg/day). Quetiapine dose was associated with higher risks of urinary retention, constipation, pneumonia, motor and thyroid disorders (1.06 ≤ OR ≤ 1.07/100 mg/day). Lithium salts dose was associated with higher risks of thyroid disorders (OR = 1.06/100 mg/day), motor disorders (OR = 1.08/100 mg/day) and delirium spectrum disorders (OR = 1.03/100 mg/day). Loxapine dose was associated with significantly higher risks of pneumonia (OR = 1.25/100 mg) and constipation (OR = 1.18/100 mg), and valproate dose was associated with significantly higher risks of electrolyte imbalances, seizures, and pneumonia (1.02 ≤ OR ≤ 1.04/100 mg/day).

## Discussion

We aimed to assess the extent to which sequences of psychotropic prescriptions contribute to the risk of in-hospital non-psychiatric adverse events in a large cohort of adults with BD and SZD. In-hospital adverse events affected 27.3% of adults aged 18–65 years with BD or SZD over 10 years. Truedson and collaborators estimated that only 1.4% of psychiatric patients are hospitalized for adverse drug events over 10 years (5), suggesting that most adverse events reported here were not precipitated by drugs.

The predictive performance was lower than that of pre-existing models designed to predict ADEs (0.63 ≤ AUCs ≤ 0.81) (26), showing that outpatient psychotropic prescription sequences are insufficient for accurately forecasting in-hospital non-psychiatric adverse events. Models that exhibited the strongest performance in predicting ADEs were trained on clinical and laboratory data (16), which are likely more informative than claims data. Even in this context, performance remains lower than that achieved by RNNs trained on healthcare datasets to predict other outcomes, such as blood glucose level in patients with diabetes (27), suggesting that ADEs are inherently more challenging to predict. Although our objective was not to build a predictive model, the poor performance of our model made it impossible to provide reliable local explanations for individual predictions. Instead, we drew general, sample-based interpretations. Such conclusions were possible because SHAP is a conservative XAI method (28), highlighting only the most robust individual associations.

The biGRU-based model outperformed the RF and XGB models, which received the same variables unordered, indicating that the orders of the prescription and hospitalization sequences participated in the prediction. This finding aligns with prior evidence that variations in medication dosage, such as the introduction of a drug, contributes to adverse event risk (13, 29). Assessing the evolution of doses over preceding months in addition to the current dose could help identify iatrogenic risk factors of in-hospital adverse events.

Psychotropic prescriptions changes over the few months preceding in-hospital adverse events — particularly urinary retention, seizures, and thyroid disorders — were more relevant to explain the development of those events. This aligns with findings that users of anticholinergic medications have a higher risk of urinary retention within 30 days of initiation than longer term users (13), that the risk of seizures is highest within 90 days of starting antipsychotics (29), and that first signs of thyroid disorders appear on average within 56.5 days of lithium salts initiation (30). In contrast, the entire sequence of psychotropic prescriptions was similarly relevant for predicting constipation, motor disorders, and cardiac rhythm or conduction disorders. This suggests that these conditions can occur any time following a prescription change. Indeed, constipation can result from both short- and long-term exposure to second-generation antipsychotics (31), and extrapyramidal syndrome can appear at highly variable intervals after antipsychotic initiation (76.5 ± 105.8 days) (32). Another possible explanation is that those events are favored by stable psychotropic prescription patterns. Therefore, the model assigns similar importance to each month, as prescriptions remain consistent across months. This may explain the findings for motor or cardiac disorders, which are strongly linked to antipsychotic use (33).

In line with previous findings, benzodiazepine use was associated with higher risk of pneumonia, in-hospital delirium, constipation, and cardiac disorders. Associations between benzodiazepines and electrolyte imbalances (34) or urinary retention (35) have only been reported in case studies, warranting further investigation of the role of GABAergic activity in such phenomena. The associations between benzodiazepines and motor disorders or seizures probably result from indication bias, as these drugs are often prescribed to manage akathisia or reduce seizure risk, revealing a limitation of the database. Another plausible explanation is that individuals with high doses of benzodiazepine have more risk of being exposed to psychotropic polypharmacy, which is associated with higher risks of adverse events. Consistent with international guidelines, our results support the cautious use of oxazepam and diazepam at the lowest possible dose (36, 37).

Antiparkinsonians, like tropatepine, have shown associations with higher risks of urinary retention, constipation or delirium spectrum disorders in previous studies, likely due to their strong anticholinergic properties. Tropatepine is primarily prescribed in BD and SZD to mitigate antipsychotic-induced parkinsonism, which may explain its association with motor disorders. Another possible explanation for this association is that tropatepine favors tardive dyskinesia (38). In line with international recommendations, our findings support the cautious use, and, if possible, deprescribing of antiparkinsonians in BD and SZD (36).

As expected, second-generation antipsychotics, lithium salts and valproate, were identified as risk factors of adverse events (5). Our results reinforce prior research showing that quetiapine is associated with higher risks of pneumonia (10), constipation (39), and urinary retention (40), which are likely due to its anticholinergic properties, as well as thyroid abnormalities (41), for which autoimmune thyroiditis is the leading pathophysiological hypothesis. Clozapine was associated with dose-dependent risks of pneumonia (10), likely related to hypersalivation induced by its cholinergic activity on M4 receptors, and constipation (31), likely driven by its anticholinergic, antiserotonin, and antihistaminic effects. While the associations between clozapine dosage and the risks of pneumonia and constipation are consistent with current prescription guidelines (42), we expected a stronger association between clozapine dosage and other adverse events, such as cardiac disorders. However, it was the first report of a risk of pneumonia associated with loxapine, an antipsychotic widely used in France. As expected, lithium salts dose was associated with higher risks of thyroid disorders. Lithium salts dose was also associated with higher risks of delirium-spectrum disorders and motor disorders, which could be explained by lithium salts-induced tremors. The pharmacological explanations for such adverse reactions are still under investigation. Valproate dose was linked to increased risks of electrolyte imbalances, likely due to hyponatremia, and pneumonia, which was only reported as significant when valproate is associated with second-generation antipsychotics (43). The mechanism of this interaction remains to be characterized. Certain psychotropic doses were among the most influential features to predict adverse events but were not significantly associated with adverse events in conditional logistic regression models. This discrepancy could be explained by a non-linear relationship between psychotropic dose and adverse event risk detected by the biGRU-based model but not the logistic regression model. Overall, prescribers should acknowledge the risks for adverse events associated with the doses of quetiapine, clozapine, loxapine, lithium salts and valproate in BD and SZD.

Hospitalization, sex, and to a lesser extent, age, were among the most influential factors in predicting adverse events. This aligns with the documented association between medical ward admission and an increased risk of rehospitalization for ADEs (44). Treatment regimens are frequently reassessed and new medications are introduced during hospitalization, increasing the risk of adverse events. Age and sex are also known to contribute to the manifestation and development of psychotropic adverse effects in BD and SZD.

Overall, results were globally consistent between SHAP values analyses and logistic regression analyses. Additionally, some findings were consistent with current knowledge on psychotropics, further supporting the application of *post-hoc* XAI to a neural network model processing prescription sequences to obtain a valid, automated, data-driven assessment of iatrogenic risks. Our model could be used retrospectively to generate hypotheses on the iatrogenic risk factors of an adverse event in clinical settings. Besides, we ensured our model required minimal computing resources, enabling local deployment thereby ensuring that sensitive patient information remains within the care facility. However, our findings also suggest that the prediction accuracy for adverse events from a model integrated into prescription software would be limited. A clinical decision support system designed to support treatment adjustments would require more than just past psychotropic prescription sequences and demographic information to provide reliable recommendations.

Our study presented several limitations. Some of our results were very likely explained by an indication bias: for instance, the significant association between the dose of valproate, an antiepileptic, and in-hospital seizure may be explained by the fact that patients at higher risk of seizures are more likely to be prescribed higher dose of valproate. We used data on the reimbursement of medication rather than its actual consumption. We may therefore have overestimated exposure to psychotropics as 20.8% of individuals with BD or SZD adhere poorly to prescribed treatments in outpatient settings (45). Outcome events were mainly collected based on inpatient discharge diagnoses that may be affected by underreporting or coding inaccuracies (46), which might have affected the results. Besides, we did not investigate medication practices after the events, questioning the semiologic imputability of those events. Our models did not account for how adverse events might contribute to the development of subsequent adverse events. For instance, constipation can predispose individuals to urinary retention, potentially mediating some of the effects of psychotropics on urinary retention. Additionally, we did not match case and control individuals on somatic comorbidities not included in the Charlson comorbidity index, which might limit comparability between the individuals. Our database lacked several clinically relevant information, such as body mass index, smoking status or indicators of renal function, which may mediate or confound the effects of psychotropics on the events. A higher proportion of our sample had a non-zero Charlson Comorbidity Index (44.9%) compared to the general adult population (29.6%) (47). This discrepancy may limit the generalizability of the results to the general adult population with BD or SZD. Additionally, the greater frailty may have participated in the underestimation of our risk estimates. Finally, SHAP values for neural networks are likely sensitive to noise due to the stochastic nature of the gradient. We partially addressed this issue by focusing on the top 10 features with the highest absolute SHAP values. While this approach prioritized the most significant factors, it may have led to the omission of less prominent but potentially interesting associations, which could be valuable for pharmacovigilance. Although Shapley values have been identified as the most effective post-hoc, model-agnostic method for explainability (19, 24), less computationally expensive and time-consuming XAI methods exist and may also provide valuable insights (48).

## Data Availability

The data analyzed in this study is subject to the following licenses/restrictions: Datasets cannot be made publicly available as access is restricted to authorized individuals only. Requests to access these datasets should be directed to https://www.snds.gouv.fr/SNDS/Processus-d-acces-aux-donnees.
