# Supplementary material for: Neural network analysis of the contribution of psychotropic prescription sequences to the risk of non-psychiatric adverse events in bipolar and schizophrenia spectrum disorders
Source: Front Digit Health. 2025 Sep 4;7:1633220. doi: 10.3389/fdgth.2025.1633220 (PMC12443787; doi:10.3389/fdgth.2025.1633220)
Supplement: Supplementary file 1 [file Datasheet1.docx]

**Supplementary Information**

Neural Network Analysis of the Contribution of Psychotropic Prescription Sequences to the Risk of non-psychiatric Adverse Events in Bipolar and Schizophrenia Spectrum Disorders

## **SM1: Prescription duration.**

We defined the maximum prescription durations based on the type of packaging dispensed:

- Medications for durations of fewer than 28 days. These were estimated to last an average of 14 days and typically included packages containing 5–15 pills or injectable forms (e.g., fluphenazine and risperidone depot injections). The estimated interval between deliveries ranged from 7 to 21 days, with a 7-day grace period. If no subsequent delivery was recorded within 21 days of the initial delivery or if no repeat delivery occurred before December 31, 2022, the dose was considered missing. To prevent overestimation of doses due to stockpiling, we assumed that medications were consumed at the average dose over 14 days when the duration between deliveries lasted fewer than 7 days.
- Medications for durations of 28–30 days. These included packages containing more than 20 pills, as French pharmacists are legally restricted to dispensing a maximum of 30 days' treatment at a time (per Article R. 5132-12 of the French Public Health Code). The estimated interval between deliveries ranged from 21 to 35 days, with a 7-day grace period. If no subsequent delivery was recorded within 35 days of the initial delivery, if the delivery occurred in December 2022, or if no repeat delivery occurred before December 31, 2022, the dose was considered missing. To avoid artificially inflating dose values due to stockpiling, we assumed medications were consumed at the average dose over 28 days when the duration between deliveries lasted fewer than 21 days.

## **SM2: Alternative models**

- Random Forest (RF) estimates multiple decision trees by imposing a series of conditions on the input to predict the output. The trees are trained in parallel on different subsets of data and each tree produces a binary prediction: the final prediction for the output is found by majority vote. RF has a good explainability and transparency. We set one hyperparameter: the maximal number of features in each tree.
- Extreme gradient boosting (XGB) (Chen and Guestrin, 2016) creates an ensemble of decision trees through an iterative process. Unlike Random Forest, XGB builds trees sequentially, with each new tree focusing on minimizing the residual errors of the combined previous trees. XGB then applies several regularization techniques to limit overfitting. XGB is less interpretable than RF but has demonstrated better performance (Sahin, 2020). We adjusted three hyperparameters: the number of gradient boosted trees, the learning rate, the maximum tree depth for base learners, and the weights to balance the cases.

**Figure S1. Prediction loss during training of the biGRU-based model with a 16-dimension hidden layer in the training and validation subsets at each epoch in one fold of the cross-validation.**
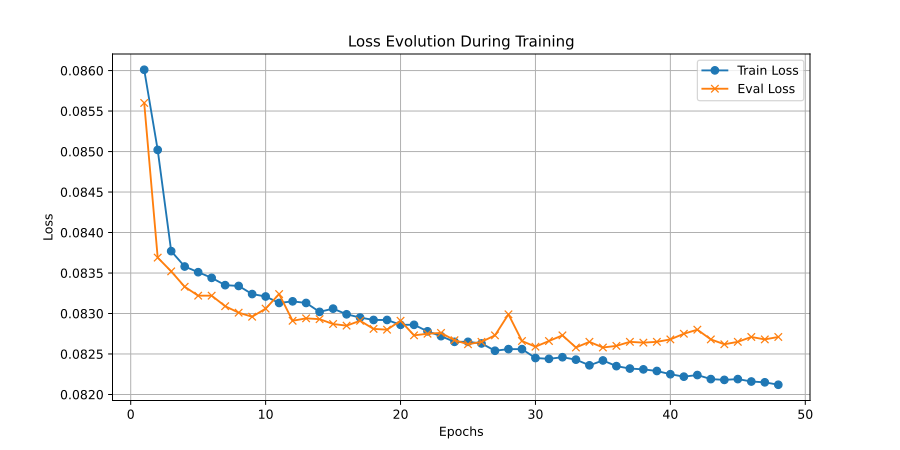


**Figure S2. Mean SHAP values for each feature of one individual across all months when predicting urinary retention using the biGRU-based model.** Red color represents positive SHAP values and blue color represents negative SHAP values. The reference of expected SHAP value is set to 0, i.e., no contribution from the individual’s features to the probability of urinary retention. In this example, tropatepine dose had the highest absolute SHAP value, contributing the most to the patient’s total SHAP value. Besides, the patient’s total SHAP value was positive (21.667%), indicating that this combination of feature values increased the probability of predicting urinary retention.


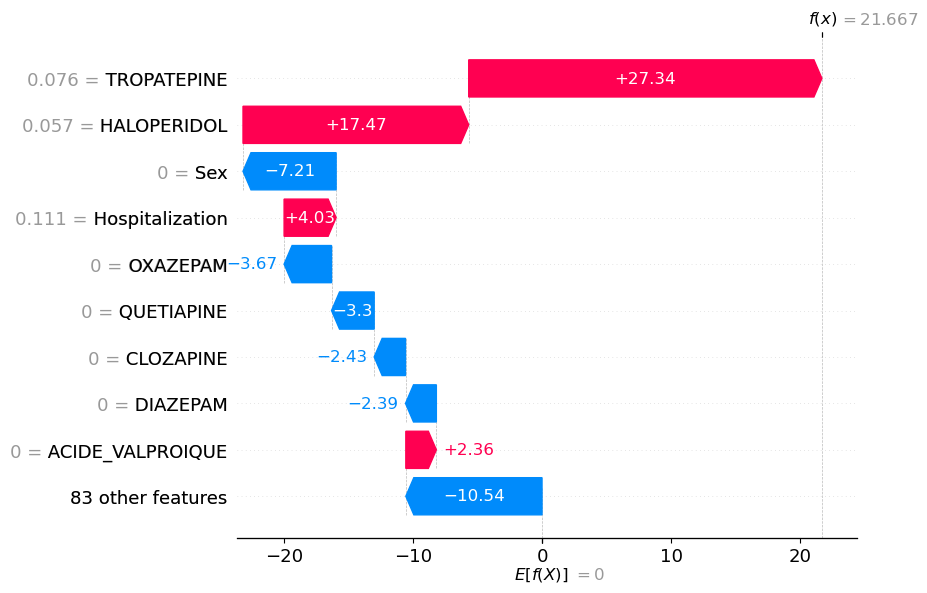


**Table S1. ICD-10 diagnosis codes used to define the target population.**

| Disorders | ICD-10 code | Description |
| --- | --- | --- |
| Schizophrenia spectrum disorders | F20.0 | Paranoid schizophrenia |
|  | F20.1 | Hebephrenic schizophrenia |
|  | F20.2 | Catatonic schizophrenia |
|  | F20.3 | Undifferentiated schizophrenia |
|  | F20.4 | Post-schizophrenic depression |
|  | F20.5 | Residual schizophrenia |
|  | F20.6 | Simple schizophrenia |
|  | F20.8 | Other schizophrenia |
|  | F20.9 | Schizophrenia, unspecified |
|  | F21 | Schizotypal disorder |
|  | F22 | Persistent delusional disorders |
|  | F23 | Acute and transient psychotic disorders |
|  | F24 | Shared psychotic disorder |
|  | F25 | Schizoaffective disorders |
|  | F28 | Other nonorganic psychotic disorders |
|  | F29 | Unspecified nonorganic psychosis |
| Bipolar disorders | F30 | Manic episode |
|  | F31 | Bipolar disorders |

**Table S2. Definition of the studied in-hospital adverse events.**

| **Adverse event** | **Definition** |
| --- | --- |
| Constipation | K59.0, K56.0, R10.9, K56.6, K56.7 [(Cai et al., 2014)](https://www.zotero.org/google-docs/?7Wzkef)  *Procedures:* HJGD001, ZCQK004, ZCQK002 |
| Urinary retention | R33, R39.1 [(Stephenson et al., 2011)](https://www.zotero.org/google-docs/?Adt65V)  *Procedures:* JDJD001, JDLF001, JDQM001 |
| Cardiac rhythm or conduction disorders | I47, I49 [(Tamariz et al., 2012)](https://www.zotero.org/google-docs/?WACLDL), I45 |
| Electrolyte imbalances | E87 |
| Pneumonia | J12, J13, J15, J18, J69 [(Kuo et al., 2013)](https://www.zotero.org/google-docs/?MLurbh) |
| Seizures | G40, O99.350, R56.9 [(Chui et al., 2016)](https://www.zotero.org/google-docs/?3F2Hej) |
| Motor disorders | G21.1, G21.2, G21.8, G21.9, G24, G25, R25 [(Kadakia et al., 2022)](https://www.zotero.org/google-docs/?zwNQL1) |
| Delirium spectrum disorders | F05 except F05.1, R41, G92 except G93.40 and G93.49 |
| Thyroid disorders | E0 except E00 [(Huang et al., 2022)](https://www.zotero.org/google-docs/?hgZHWN), E21 |

##

**Table S3. Definition of the studied comorbidities.**

| Comorbidity | Comorbidity definition period | ICD-10 codes and/or medical procedures and/or treatments |
| --- | --- | --- |
| Used to measure the Charlson Comorbidity Index | | |
| Diabetes | From 12 months before the study period to the end of the study period [(Bannay et al., 2016)](https://www.zotero.org/google-docs/?o8DIwk) (2012 to 2022) | E10.0, E10.1, E10.6, E10.8, E10.9, E11.0, E11.1, E11.6, E11.8, E11.9, E12.0, E12.1, E12.6, E12.8-E13.1, E13.6, E13.8-E14.1, E14.6, E14.8, E14.9 [(Bannay et al., 2016)](https://www.zotero.org/google-docs/?wxIews)  **AND/OR**  At least 3 reimbursements of oral antidiabetic agents and/or insulin: ATC codes A10AB01, A10AB03, A10AB04, A10AB05, A10AB06, A10AC01, A10AC03, A10AC04, A10AD01, A10AD03, A10AD04, A10AD05, A10AE01, A10AE02, A10AE03, A10AE04, A10AE05, A10AE30, A10BA02, A10BB01, A10BB03, A10BB04, A10BB06, A10BB07, A10BB09, A10BB12, A10BD02, A10BD03, A10BD05, A10BD07, A10BD08, A10BD10, A10BD15, A10BD16, A10BF01, A10BF02, A10BG02, A10BG03, A10BH01, A10BH02, A10BH03, A10BX02, A10BX04, A10BX07, A10BX09, A10BX10, A10BX11, A10BX12 [(Quantin et al., 2015)](https://www.zotero.org/google-docs/?av3hGE) |
| Myocardial infarction |  | I21, I22, I25.2, I25.5 [(Bannay et al., 2016)](https://www.zotero.org/google-docs/?B31HoQ) |
| Moderate or severe renal disease |  | I12.0, I13.1, N03.2-N03.7, N05.2-N05.7, N18, N19, N25.0, Z49.0, Z49.1, Z49.2, Z94.0, Z99.2 [(Bannay et al., 2016)](https://www.zotero.org/google-docs/?4IAy8R)  **AND/OR**  Procedures: dialysis (see the algorithm in [(Quantin et al., 2015)](https://www.zotero.org/google-docs/?e9wWOu)) |
| Diabetes with end-organ damage |  | E10.2-E10.5, E10.7, E11.2-E11.5, E11.7, E12.2-E12.5, E12.7, E13.2-E13.5, E13.7, E14.2-E14.5, E14.7  **AND/OR**  Diabetes and at least one comorbid condition from the following list: myocardial infarction, moderate or severe renal disease, or cerebrovascular disease  **AND/OR**  Procedures (CCAM codes): Laser surgery for diabetic retinopathy (BGJA001, BGFA009, BGDA008, BGDA006, BGDA005, BGDA004, BGDA003, BGDA002, BGDA001) [(Bannay et al., 2016)](https://www.zotero.org/google-docs/?Cbst5C) |
| Congestive heart failure |  | I11.0, I13.0, I13.2, I50 [(Bannay et al., 2016)](https://www.zotero.org/google-docs/?VAEwod) |
| Ulcer disease |  | K25-K28 [(Bannay et al., 2016)](https://www.zotero.org/google-docs/?NevYLx) |
| Any tumor (including lymphoma and leukemia except for malignant neoplasm of skin) |  | [(Bannay et al., 2016)](https://www.zotero.org/google-docs/?zWI6UF) C00-C26, C30-C34, C37-C41, C43, C45-C58, C60- C76, C81-C85, C88, C90-C97 |
| Metastatic solid tumor |  | [(Bannay et al., 2016)](https://www.zotero.org/google-docs/?Z0XS2v) C77-C80 |
| Mild liver disease |  | [(Bannay et al., 2016)](https://www.zotero.org/google-docs/?esclb3) B18, K70.0-K70.3, K70.9, K71.3, K71.5, K71.7, K73, K74, K76.0, K76.2-K76.4, K76.8, K76.9, Z94.4 |
| Moderate or severe liver disease |  | [(Bannay et al., 2016)](https://www.zotero.org/google-docs/?gPFxO6) I85.0, I85.9, I86.4, I98.2, K70.4, K71.1, K72.1, K72.9, K76.5- K76.7 |
| HIV-AIDS |  | [(Bannay et al., 2016)](https://www.zotero.org/google-docs/?a1m71M) B20-B22, B24, Z21 |
| Hemiplegia |  | [(Bannay et al., 2016)](https://www.zotero.org/google-docs/?xlJ4AQ) G04.1, G11.4, G80.1, G80.2, G81, G82, G83.0, G83.1- G83.4, G83.9 |
| Connective tissue disease |  | [(Bannay et al., 2016)](https://www.zotero.org/google-docs/?y6NQhn) M05, M06, M31.5, M32, M33, M34, M35.1, M35.3, M36.0 |
| Chronic Pulmonary Disease |  | I27.8, I27.9, J40-J47, J60-J67, J68.4, J70.1, J70.3 [(Bannay et al., 2016)](https://www.zotero.org/google-docs/?Sp3jys)  **AND/OR**  [(Quantin et al., 2015)](https://www.zotero.org/google-docs/?1TpyRR) At least 2 reimbursements of bronchodilator drugs: ATC codes starting with R03 |
| Peripheral vascular disease |  | I70, I71, I73.1, I73.8, I73.9, I77.1, I79.0, I79.2, K55.1, K55.8, K55.9, Z95.8, Z95.9 [(Bannay et al., 2016)](https://www.zotero.org/google-docs/?2w4Nj3)  **AND/OR**  Procedures (CCAM codes): peripheral vascular stenting (DDAA002, DFAA002, DFAA003, DFAA004, DFAA005, DGAA002, DGAA004, DGAA005, DGAA006, DFAA003, DZSA001, EBAA002, EBFA002, EBFA006, EBFA012, EBFA016, EDAA002, EEAA002, EPCA002, EZCA004, YYYY082) |
| Other comorbidities | | |
| Traumatic brain injury | From 2012 to the end of the study period | S02.0, S02.1, S02.8, S02.91, S04.02, S04.03, S04.04, S06, S07.1, T74.4 [(Hedegaard et al., 2016)](https://www.zotero.org/google-docs/?4pFMp7) |
| Parkinson’s disease |  | [(Quantin et al., 2015)](https://www.zotero.org/google-docs/?kUpa01) G20, F02.3 |
| Dementia |  | F00-F03, F05.1, G30, G31.1 [(Bannay et al., 2016)](https://www.zotero.org/google-docs/?WBtu5l)  **AND/OR**  At least 3 reimbursements of anti-Alzheimer drugs: ATC codes starting with NO6DA, and N06DX01 [(Quantin et al., 2015)](https://www.zotero.org/google-docs/?FztCr7) |
| Multiple sclerosis |  | G35 [(Quantin et al., 2015)](https://www.zotero.org/google-docs/?LPzyue) |
| Epilepsy |  | G40, G41 [(Quantin et al., 2015)](https://www.zotero.org/google-docs/?pmCvpt) |
| Cerebrovascular disease |  | I67-69 |

*Notes. CCAM: Common Classification of Medical Acts. A complete list of existing CCAM codes can be found at* [*https://assurance-maladie.ameli.fr/etudes-et-donnees/actes-techniques-ccam*](https://assurance-maladie.ameli.fr/etudes-et-donnees/actes-techniques-ccam)*.*

**Table S4. Mean performance across the five-fold cross-validation of the models for each hyperparameter value.** The selected values for the final models are in bold. AUC corresponds to the mean AUC over the five-fold cross-validation. The hyperparameter of the RF model was *max_features* in the *RandomForestClassifier* function of *sklearn* package. The hyperparameters of the XGB model were the number of gradient boosted trees (*n_estimators* in the *XGBClassifier* function of the *xgboost* package), the learning rate (*learning_rate*), the maximum tree depth for base learners (*max_depth*), and the weights to balance the cases (*scale_pos_weight*).

| **Parameter 1** | **Value** | **Parameter 2** | **Value** | **Parameter 3** | **Value** | **Parameter 4** | **Value** | **AUC** |
| --- | --- | --- | --- | --- | --- | --- | --- | --- |
| **biGRU-based model** | | | | | | | | |
| Dimension size of the hidden layer in the GRU unit | **16** | **-** | **-** | **-** | **-** |  |  | **0.632861** |
|  | 32 | - | - | - | - |  |  | 0.631843 |
|  | 64 | - | - | - | - |  |  | 0.630553 |
| **Random forest** | | | | | | | | |
| Maximal number of predictors (max_features) | 500 | - | - | - | - |  |  | 0.499784 |
|  | 1000 | - | - | - | - |  |  | 0.499724 |
|  | **1588** | **-** | **-** | **-** | **-** |  |  | **0.499785** |
| **XGBoost** | | | | | | | | |
| Number of gradient boosted trees (n_estimators) | 50 | Boosting learning rate (learning_rate) | 0.01 | Maximum tree depth for base learners (max_depth) | 3 | Balancing of positive and negative weights (scale_pos_weight) | 1 | 0.5 |
|  |  |  |  |  |  |  | 5 | 0.5002 |
|  |  |  |  |  |  |  | 10 | 0.5122 |
|  |  |  |  |  |  |  | 20 | 0.5330 |
|  |  |  |  |  | 6 |  | 1 | 0.5 |
|  |  |  |  |  |  |  | 5 | 0.5010 |
|  |  |  |  |  |  |  | 10 | 0.5161 |
|  |  |  |  |  |  |  | 20 | 0.5362 |
|  |  |  |  |  | 8 |  | 1 | 0.5 |
|  |  |  |  |  |  |  | 5 | 0.5010 |
|  |  |  |  |  |  |  | 10 | 0.5167 |
|  |  |  |  |  |  |  | 20 | 0.5351 |
|  |  |  |  |  | 10 |  | 1 | 0.5 |
|  |  |  |  |  |  |  | 5 | 0.5009 |
|  |  |  |  |  |  |  | 10 | 0.5154 |
|  |  |  |  |  |  |  | 20 | 0.5339 |
|  |  |  | 0.05 |  | 3 |  | 1 | 0.5002 |
|  |  |  |  |  |  |  | 5 | 0.5102 |
|  |  |  |  |  |  |  | 10 | 0.5369 |
|  |  |  |  |  |  |  | 20 | 0.5362 |
|  |  |  |  |  | 6 |  | 1 | 0.5007 |
|  |  |  |  |  |  |  | 5 | 0.5120 |
|  |  |  |  |  |  |  | 10 | 0.5373 |
|  |  |  |  |  |  |  | 20 | 0.5430 |
|  |  |  |  |  | 8 |  | 1 | 0.5008 |
|  |  |  |  |  |  |  | 5 | 0.5111 |
|  |  |  |  |  |  |  | 10 | 0.5353 |
|  |  |  |  |  |  |  | 20 | 0.5422 |
|  |  |  |  |  | 10 |  | 1 | 0.5009 |
|  |  |  |  |  |  |  | 5 | 0.5117 |
|  |  |  |  |  |  |  | 10 | 0.5322 |
|  |  |  |  |  |  |  | 20 | 0.5391 |
|  |  |  | 0.1 |  | 3 |  | 1 | 0.5006 |
|  |  |  |  |  |  |  | 5 | 0.5172 |
|  |  |  |  |  |  |  | 10 | 0.5413 |
|  |  |  |  |  |  |  | 20 | 0.5418 |
|  |  |  |  |  | 6 |  | 1 | 0.5011 |
|  |  |  |  |  |  |  | 5 | 0.5165 |
|  |  |  |  |  |  |  | 10 | 0.5400 |
|  |  |  |  |  |  |  | 20 | 0.5455 |
|  |  |  |  |  | 8 |  | 1 | 0.5013 |
|  |  |  |  |  |  |  | 5 | 0.5141 |
|  |  |  |  |  |  |  | 10 | 0.5352 |
|  |  |  |  |  |  |  | 20 | 0.5423 |
|  |  |  |  |  | 10 |  | 1 | 0.5014 |
|  |  |  |  |  |  |  | 5 | 0.5126 |
|  |  |  |  |  |  |  | 10 | 0.5305 |
|  |  |  |  |  |  |  | 20 | 0.5378 |
|  | 100 |  | 0.01 |  | 3 |  | 1 | 0.5 |
|  |  |  |  |  |  |  | 5 | 0.5013 |
|  |  |  |  |  |  |  | 10 | 0.5271 |
|  |  |  |  |  |  |  | 20 | 0.5327 |
|  |  |  |  |  | 6 |  | 1 | 0.5004 |
|  |  |  |  |  |  |  | 5 | 0.5022 |
|  |  |  |  |  |  |  | 10 | 0.5299 |
|  |  |  |  |  |  |  | 20 | 0.5369 |
|  |  |  |  |  | 8 |  | 1 | 0.5004 |
|  |  |  |  |  |  |  | 5 | 0.5026 |
|  |  |  |  |  |  |  | 10 | 0.5271 |
|  |  |  |  |  |  |  | 20 | 0.5379 |
|  |  |  |  |  | 10 |  | 1 | 0.5004 |
|  |  |  |  |  |  |  | 5 | 0.5031 |
|  |  |  |  |  |  |  | 10 | 0.5310 |
|  |  |  |  |  |  |  | 20 | 0.5395 |
|  |  |  | 0.05 |  | 3 |  | 1 | 0.5006 |
|  |  |  |  |  |  |  | 5 | 0.5165 |
|  |  |  |  |  |  |  | 10 | 0.5408 |
|  |  |  |  |  |  |  | 20 | 0.5421 |
|  |  |  |  |  | 6 |  | 1 | 0.5010 |
|  |  |  |  |  |  |  | 5 | 0.5157 |
|  |  |  |  |  |  |  | 10 | 0.5390 |
|  |  |  |  |  |  |  | 20 | 0.5445 |
|  |  |  |  |  | 8 |  | 1 | 0.5010 |
|  |  |  |  |  |  |  | 5 | 0.5134 |
|  |  |  |  |  |  |  | 10 | 0.5356 |
|  |  |  |  |  |  |  | 20 | 0.5418 |
|  |  |  |  |  | 10 |  | 1 | 0.5013 |
|  |  |  |  |  |  |  | 5 | 0.5117 |
|  |  |  |  |  |  |  | 10 | 0.5298 |
|  |  |  |  |  |  |  | 20 | 0.5383 |
|  |  |  | 0.1 |  | 3 |  | 1 | 0.5011 |
|  |  |  |  |  |  |  | 5 | 0.5196 |
|  |  |  |  |  |  |  | 10 | 0.5446 |
|  |  |  |  |  |  |  | 20 | 0.5482 |
|  |  |  |  |  | 6 |  | 1 | 0.5016 |
|  |  |  |  |  |  |  | 5 | 0.5174 |
|  |  |  |  |  |  |  | 10 | 0.5385 |
|  |  |  |  |  |  |  | 20 | 0.5439 |
|  |  |  |  |  | 8 |  | 1 | 0.5017 |
|  |  |  |  |  |  |  | 5 | 0.5142 |
|  |  |  |  |  |  |  | 10 | 0.5319 |
|  |  |  |  |  |  |  | 20 | 0.5389 |
|  |  |  |  |  | 10 |  | 1 | 0.5016 |
|  |  |  |  |  |  |  | 5 | 0.5115 |
|  |  |  |  |  |  |  | 10 | 0.5266 |
|  |  |  |  |  |  |  | 20 | 0.5339 |
|  | **200** |  | 0.01 |  | 3 |  | 1 | 0.5002 |
|  |  |  |  |  |  |  | 5 | 0.5070 |
|  |  |  |  |  |  |  | 10 | 0.5355 |
|  |  |  |  |  |  |  | 20 | 0.5345 |
|  |  |  |  |  | 6 |  | 1 | 0.5007 |
|  |  |  |  |  |  |  | 5 | 0.5093 |
|  |  |  |  |  |  |  | 10 | 0.5367 |
|  |  |  |  |  |  |  | 20 | 0.5413 |
|  |  |  |  |  | 8 |  | 1 | 0.5006 |
|  |  |  |  |  |  |  | 5 | 0.5086 |
|  |  |  |  |  |  |  | 10 | 0.5346 |
|  |  |  |  |  |  |  | 20 | 0.5428 |
|  |  |  |  |  | 10 |  | 1 | 0.5007 |
|  |  |  |  |  |  |  | 5 | 0.5078 |
|  |  |  |  |  |  |  | 10 | 0.5314 |
|  |  |  |  |  |  |  | 20 | 0.5396 |
|  |  |  | 0.05 |  | 3 |  | 1 | 0.5011 |
|  |  |  |  |  |  |  | 5 | 0.5197 |
|  |  |  |  |  |  |  | 10 | 0.5445 |
|  |  |  |  |  |  |  | 20 | 0.5473 |
|  |  |  |  |  | 6 |  | 1 | 0.5015 |
|  |  |  |  |  |  |  | 5 | 0.5164 |
|  |  |  |  |  |  |  | 10 | 0.5382 |
|  |  |  |  |  |  |  | 20 | 0.5443 |
|  |  |  |  |  | 8 |  | 1 | 0.5014 |
|  |  |  |  |  |  |  | 5 | 0.5132 |
|  |  |  |  |  |  |  | 10 | 0.5329 |
|  |  |  |  |  |  |  | 20 | 0.5398 |
|  |  |  |  |  | 10 |  | 1 | 0.5015 |
|  |  |  |  |  |  |  | 5 | 0.5109 |
|  |  |  |  |  |  |  | 10 | 0.5266 |
|  |  |  |  |  |  |  | 20 | 0.5340 |
|  |  |  | **0.1** |  | **3** |  | 1 | 0.5014 |
|  |  |  |  |  |  |  | 5 | 0.5211 |
|  |  |  |  |  |  |  | 10 | 0.5445 |
|  |  |  |  |  |  |  | **20** | **0.5502** |
|  |  |  |  |  | 6 |  | 1 | 0.5021 |
|  |  |  |  |  |  |  | 5 | 0.5160 |
|  |  |  |  |  |  |  | 10 | 0.5347 |
|  |  |  |  |  |  |  | 20 | 0.5404 |
|  |  |  |  |  | 8 |  | 1 | 0.5021 |
|  |  |  |  |  |  |  | 5 | 0.5123 |
|  |  |  |  |  |  |  | 10 | 0.5271 |
|  |  |  |  |  |  |  | 20 | 0.5325 |
|  |  |  |  |  | 10 |  | 1 | 0.5022 |
|  |  |  |  |  |  |  | 5 | 0.5093 |
|  |  |  |  |  |  |  | 10 | 0.5200 |
|  |  |  |  |  |  |  | 20 | 0.5255 |
|  | 500 |  | 0.01 |  | 3 |  | 1 | 0.5006 |
|  |  |  |  |  |  |  | 5 | 0.5163 |
|  |  |  |  |  |  |  | 10 | 0.5412 |
|  |  |  |  |  |  |  | 20 | 0.5413 |
|  |  |  |  |  | 6 |  | 1 | 0.5010 |
|  |  |  |  |  |  |  | 5 | 0.5154 |
|  |  |  |  |  |  |  | 10 | 0.5394 |
|  |  |  |  |  |  |  | 20 | 0.5455 |
|  |  |  |  |  | 8 |  | 1 | 0.5012 |
|  |  |  |  |  |  |  | 5 | 0.5128 |
|  |  |  |  |  |  |  | 10 | 0.5353 |
|  |  |  |  |  |  |  | 20 | 0.5421 |
|  |  |  |  |  | 10 |  | 1 | 0.5011 |
|  |  |  |  |  |  |  | 5 | 0.5107 |
|  |  |  |  |  |  |  | 10 | 0.5301 |
|  |  |  |  |  |  |  | 20 | 0.5384 |
|  |  |  | 0.05 |  | 3 |  | 1 | 0.5014 |
|  |  |  |  |  |  |  | 5 | 0.5214 |
|  |  |  |  |  |  |  | 10 | 0.5451 |
|  |  |  |  |  |  |  | 20 | 0.5499 |
|  |  |  |  |  | 6 |  | 1 | 0.5021 |
|  |  |  |  |  |  |  | 5 | 0.5148 |
|  |  |  |  |  |  |  | 10 | 0.5332 |
|  |  |  |  |  |  |  | 20 | 0.5388 |
|  |  |  |  |  | 8 |  | 1 | 0.5018 |
|  |  |  |  |  |  |  | 5 | 0.5101 |
|  |  |  |  |  |  |  | 10 | 0.5245 |
|  |  |  |  |  |  |  | 20 | 0.5310 |
|  |  |  |  |  | 10 |  | 1 | 0.5019 |
|  |  |  |  |  |  |  | 5 | 0.5079 |
|  |  |  |  |  |  |  | 10 | 0.5180 |
|  |  |  |  |  |  |  | 20 | 0.5219 |
|  |  |  | 0.1 |  | 3 |  | 1 | 0.5019 |
|  |  |  |  |  |  |  | 5 | 0.5210 |
|  |  |  |  |  |  |  | 10 | 0.5416 |
|  |  |  |  |  |  |  | 20 | 0.5476 |
|  |  |  |  |  | 6 |  | 1 | 0.5023 |
|  |  |  |  |  |  |  | 5 | 0.5122 |
|  |  |  |  |  |  |  | 10 | 0.5246 |
|  |  |  |  |  |  |  | 20 | 0.5296 |
|  |  |  |  |  | 8 |  | 1 | 0.5023 |
|  |  |  |  |  |  |  | 5 | 0.5081 |
|  |  |  |  |  |  |  | 10 | 0.5162 |
|  |  |  |  |  |  |  | 20 | 0.5177 |
|  |  |  |  |  | 10 |  | 1 | 0.5024 |
|  |  |  |  |  |  |  | 5 | 0.5057 |
|  |  |  |  |  |  |  | 10 | 0.5090 |
|  |  |  |  |  |  |  | 20 | 0.5113 |

**Table S5. Performance of the models in the test set.**

| **Model** | **AUC** | | | **Sensitivity** | | | **Specificity** | | | **Precision** | | |
| --- | --- | --- | --- | --- | --- | --- | --- | --- | --- | --- | --- | --- |
|  | biGRU-based model | RF | XGB | biGRU-based model | RF | XGB | biGRU-based model | RF | XGB | biGRU-based model | RF | XGB |
| Urinary retention | 0.60 | 0.5 | 0.57 | 0.62 | 0 | 0.64 | 0.58 | 1 | 0.49 | 0.09 | 0.04 | 0.07 |
| Constipation | 0.58 | 0.5 | 0.5 | 0.61 | 0 | 0.99 | 0.55 | 1 | 0.01 | 0.19 | 0.06 | 0.15 |
| Electrolyte imbalances | 0.58 | 0.5 | 0.5 | 0.64 | 0 | 0.98 | 0.53 | 1 | 0.02 | 0.15 | 0.05 | 0.11 |
| Motor disorders | 0.57 | 0.5 | 0.53 | 0.54 | 0 | 0.08 | 0.60 | 1 | 0.98 | 0.04 | 0.14 | 0.09 |
| Delirium spectrum disorders | 0.59 | 0.5 | 0.59 | 0.70 | 0 | 0.63 | 0.48 | 1 | 0.54 | 0.07 | 0.08 | 0.08 |
| Seizures | 0.67 | 0.5 | 0.63 | 0.65 | 0 | 0.29 | 0.69 | 1 | 0.96 | 0.05 | 0 | 0.15 |
| Pneumonia | 0.61 | 0.5 | 0.53 | 0.65 | 0 | 0.94 | 0.57 | 1 | 0.12 | 0.13 | 0.03 | 0.10 |
| Cardiac rhythm or conduction disorders | 0.55 | 0.5 | 0.5 | 0.57 | 0 | 0.02 | 0.54 | 1 | 0.99 | 0.04 | 0.04 | 0.04 |
| Thyroid disorders | 0.64 | 0.5 | 0.65 | 0.64 | 0 | 0.81 | 0.64 | 1 | 0.48 | 0.12 | 0.05 | 0.11 |

**Table S6. Results of the conditional logistic regression analyses of the risks of adverse events with psychotropic medications as predictors.** Results were obtained within strata of matched case and control individuals.

| **Urinary retention** | | | **Constipation** | | | **Electrolyte imbalances** | | |
| --- | --- | --- | --- | --- | --- | --- | --- | --- |
| Drug | OR (95% CI) | *p* | Drug | OR (95% CI) | *p* | Drug | OR (95% CI) | *p* |
| Olanzapine (/10 mg) | 1.07 (0.93: 1.21) | 0.327 | **Loxapine (/100 mg)** | **1.18 (1.08: 1.29)** | **0.001** | Risperidone | 1.01 (0.99: 1.04) | 0.306 |
| Valproate (/100 mg) | 1.00 (0.98: 1.01) | 0.484 | Olanzapine (/10 mg) | 1.02 (0.90: 1.11) | 0.589 | **Tropatepine** | **1.01 (1.01: 1.02)** | **0.036** |
| Lithium salts(/100 mg) | 1.00 (0.97: 1.02) | 0.759 | **Clozapine (/10 mg)** | **1.01 (1.01: 1.02)** | **<0.001** | **Diazepam** | **1.03 (1.02: 1.04)** | **<0.001** |
| **Diazepam** | **1.04 (1.02: 1.05)** | **<0.001** | **Oxazepam (/10 mg)** | **1.03 (1.02: 1.04)** | **<0.001** | **Oxazepam (/10 mg)** | **1.04 (1.03: 1.06)** | **<0.001** |
| **Quetiapine (/100 mg)** | **1.07 (1.03: 1.12)** | **0.001** | Lithium salts (/100 mg) | 1.00 (0.99: 1.00) | 0.147 | **Valproate (/100 mg)** | **1.02 (1.01: 1.03)** | **<0.001** |
| **Tropatepine** | **1.03 (1.02: 1.04)** | **<0.001** | **Quetiapine (/100 mg)** | **1.07 (1.05: 1.10)** | **<0.001** | - | - | - |
| - | - | - | **Diazepam** | **1.02 (1.01: 1.02)** | **<0.001** | - | - | - |
| - | - | - | **Tropatepine** | **1.03 (1.02: 1.04)** | **<0.001** | - | - | - |
| **Motor disorders** | | | **Delirium spectrum disorders** | | | **Seizures** | | |
| Drug | OR (95% CI) | *p* | Drug | OR (95% CI) | *p* | Drug | OR (95% CI) | *p* |
| Aripiprazole | 0.99 (0.97: 1.01) | 0.257 | Loxapine (/100 mg) | 1.08 (0.88: 1.28) | 0.417 | Risperidone | 0.96 (0.92: 1.01) | 0.140 |
| **Oxazepam (/10 mg)** | **1.04 (1.01: 1.07)** | **0.006** | Quetiapine (/100 mg) | 1.03 (0.99: 1.07) | 0.113 | Aripiprazole | 0.99 (0.97: 1.01) | 0.135 |
| **Quetiapine (/100 mg)** | **1.07 (1.00: 1.14)** | **0.042** | **Oxazepam (/10 mg)** | **1.03 (1.02: 1.05)** | **<0.001** | **Diazepam** | **1.03 (1.01: 1.04)** | **0.006** |
| **Diazepam** | **1.03 (1.01: 1.05)** | **0.001** | **Diazepam** | **1.03 (1.01: 1.04)** | **<0.001** | **Oxazepam (/10 mg)** | **1.05 (1.02: 1.07)** | **<0.001** |
| **Tropatepine** | **1.05 (1.03: 1.07)** | **<0.001** | **Tropatepine** | **1.03 (1.02: 1.05)** | **<0.001** | Lithium salts (/100 mg) | 0.98 (0.94: 1.03) | 0.467 |
| **Lithium salts (/100 mg)** | **1.08 (1.04: 1.11)** | **<0.001** | **Lithium salts (/100 mg)** | **1.03 (1.01: 1.05)** | **0.011** | **Valproate (/100 mg)** | **1.04 (1.02: 1.06)** | **<0.001** |
| **Pneumonia** | | | **Cardiac rythme or conduction disorders** | | | **Thyroid disorders** | | |
| Drug | OR (95% CI) | *p* | Drug | OR (95% CI) | *p* | Drug | OR (95% CI) | *p* |
| **Quetiapine (/100 mg)** | **1.05 (1.02: 1.09)** | **0.002** | Loxapine  (/100 mg) | 1.19 (0.95: 1.43) | 0.123 | **Quetiapine**  **(/100 mg)** | **1.06 (1.03: 1.10)** | **0.001** |
| **Loxapine (/100 mg)** | **1.25 (1.11: 1.40)** | **<0.001** | Risperidone | 0.97 (0.93: 1.01) | 0.161 | Oxazepam  (/10 mg) | 1.00 (0.98: 1.01) | 0.821 |
| **Valproate (/100 mg)** | **1.02 (1.01: 1.03)** | **<0.001** | Tropatepine | 1.00 (0.98: 1.02) | 0.783 | Valproate  (/100 mg) | 1.01 (0.99: 1.02) | 0.086 |
| Tropatepine | 1.01 (1.00: 1.02) | 0.231 | **Oxazepam (/10 mg)** | **1.03 (1.01: 1.06)** | **0.008** | **Aripiprazole** | **1.01 (1.01: 1.02)** | **0.027** |
| **Oxazepam (/10 mg)** | **1.05 (1.04: 1.07)** | **<0.001** | **Diazepam** | **1.02 (1.01: 1.03)** | **0.012** | **Lithium salts**  **(/100 mg)** | **1.06 (1.05: 1.08)** | **<0.001** |
| **Diazepam** | **1.03 (1.02: 1.04)** | **<0.001** | - | - | - | - | - | - |

# References

[Bannay, A., Chaignot, C., Blotière, P.-O., Basson, M., Weill, A., Ricordeau, P., & Alla, F. (2016). The Best Use of the Charlson Comorbidity Index With Electronic Health Care Database to Predict Mortality. *Medical Care*, *54*(2), 188‑194. https://doi.org/10.1097/MLR.0000000000000471](https://www.zotero.org/google-docs/?oxtfYj)

[Cai, Q., Buono, J. L., Spalding, W. M., Sarocco, P., Tan, H., Stephenson, J. J., Carson, R. T., & Doshi, J. A. (2014). Healthcare costs among patients with chronic constipation : A retrospective claims analysis in a commercially insured population. *Journal of Medical Economics*, *17*(2), 148‑158. https://doi.org/10.3111/13696998.2013.860375](https://www.zotero.org/google-docs/?oxtfYj)

[Chui, C. S. L., Chan, E. W., Wong, A. Y. S., Root, A., Douglas, I. J., & Wong, I. C. K. (2016). Association between oral fluoroquinolones and seizures : A self-controlled case series study. *Neurology*, *86*(18), 1708‑1715. https://doi.org/10.1212/WNL.0000000000002633](https://www.zotero.org/google-docs/?oxtfYj)

[Hedegaard, H., Johnson, R. L., Warner, M., Chen, L.-H., & Annest, J. L. (2016). Proposed Framework for Presenting Injury Data Using the International Classification of Diseases, Tenth Revision, Clinical Modification (ICD-10-CM)  Diagnosis Codes. *National Health Statistics Reports*, *89*, 1‑20.](https://www.zotero.org/google-docs/?oxtfYj)

[Huang, S.-C., Gau, S.-Y., Huang, J.-Y., Wu, W.-J., & Wei, J. C.-C. (2022). Increased Risk of Hypothyroidism in People with Asthma : Evidence from a Real-World Population-Based Study. *Journal of Clinical Medicine*, *11*(10). https://doi.org/10.3390/jcm11102776](https://www.zotero.org/google-docs/?oxtfYj)

[Kadakia, A., Brady, B. L., Dembek, C., Williams, G. R., & Kent, J. M. (2022). The incidence and economic burden of extrapyramidal symptoms in patients with schizophrenia treated with second generation antipsychotics in a Medicaid  population. *Journal of Medical Economics*, *25*(1), 87‑98. https://doi.org/10.1080/13696998.2021.2019501](https://www.zotero.org/google-docs/?oxtfYj)

[Kuo, C.-J., Yang, S.-Y., Liao, Y.-T., Chen, W. J., Lee, W.-C., Shau, W.-Y., Chang, Y.-T., Tsai, S.-Y., & Chen, C.-C. (2013). Second-generation antipsychotic medications and risk of pneumonia in schizophrenia. *Schizophrenia Bulletin*, *39*(3), 648‑657. https://doi.org/10.1093/schbul/sbr202](https://www.zotero.org/google-docs/?oxtfYj)

[Quantin, C., Roussot, A., Cottenet, J., & Besson, J. (2015). *Méthode de la cartographie des pathologies et des dépenses de l’Assurance Maladie*. https://www.assurance-maladie.ameli.fr/etudes-et-donnees/par-theme/pathologies/cartographie-assurance-maladie/methode-cartographie-pathologies-depenses-assurance-maladie#text_155397](https://www.zotero.org/google-docs/?oxtfYj)

[Stephenson, A., Seitz, D., Bell, C. M., Gruneir, A., Gershon, A. S., Austin, P. C., Fu, L., Anderson, G. M., Rochon, P. A., & Gill, S. S. (2011). Inhaled anticholinergic drug therapy and the risk of acute urinary retention in chronic obstructive pulmonary disease : A population-based study. *Archives of Internal Medicine*, *171*(10), 914‑920. https://doi.org/10.1001/archinternmed.2011.170](https://www.zotero.org/google-docs/?oxtfYj)

[Tamariz, L., Harkins, T., & Nair, V. (2012). A systematic review of validated methods for identifying ventricular arrhythmias using administrative and claims data. *Pharmacoepidemiology and Drug Safety*, *21 Suppl 1*, 148‑153. https://doi.org/10.1002/pds.2340](https://www.zotero.org/google-docs/?oxtfYj)
